# Accelerating Variable Cell Shape Molecular Dynamics with a Position-Dependent Mass Matrix

**DOI:** 10.1021/acs.jctc.5c00237

**Published:** 2025-06-13

**Authors:** Martin Sommer-Jörgensen, Marco Krummenacher, Stefan Goedecker

**Affiliations:** Department of Physics, 27209 University of Basel, Klingelbergstrasse 82, Basel CH-4056, Switzerland

## Abstract

In molecular dynamics (MD), the accessible time scales are limited by the necessity to choose sufficiently small time steps so that the fastest vibrations of the system can still be modeled. Mass tensor molecular dynamics (MTMD) aims to increase the time step by augmenting the Hamiltonian with a position-dependent mass matrix. Higher masses are assigned to modes with fast vibrations. These modes are identified by using an approximate Hessian matrix. The approximate Hessian matrix presented in this paper is applicable to the simulation of molecular systems, where no changes in the bonding pattern occur. We have adapted the MTMD method to variable cell shape systems and present a suitable symplectic integrator. The efficiency of the method is demonstrated for a system of molecular crystals consisting of *N*-(4-Methylbenzylidene)-4-methylaniline, where we could sample transitions between two polymorphs and thereby increase the time step by a factor of 4.4 to speed up the simulation. We have also simulated liquid water at the density function theory level, where we have achieved an acceleration by a factor of 2.8.

## Graphical abstract

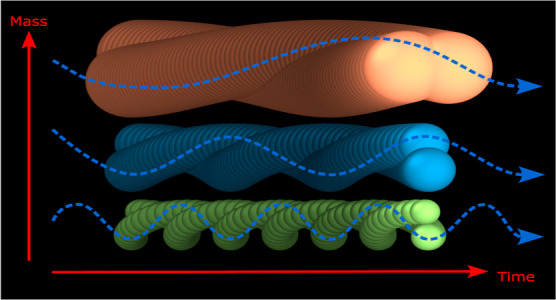

## 1 Introduction

A position-dependent mass matrix in molecular dynamics (MD) was introduced by Bennet^1^ and further examined by Tsuchida.^2^ The resulting method is called mass tensor molecular dynamics (MTMD), where the mass matrix $M\left(q\right)\in R^{3N\times 3N}$ is generalized to be a function of the configurational coordinates ***q***. The freedom to choose an arbitrary function *M*(***q***) can be used to slow down certain vibrational modes of the system and thus increase the time step in MD simulations. Unlike other common techniques to increase the MD time step, no constraints, e.g. on bond lengths and bond angles, are imposed. Tsuchida noticed that the molecular dynamics simulation with a position-dependent mass matrix reproduces the probability density of the canonical ensemble as long as the determinant of the mass matrix is restricted to be constant. We repeat this derivation for a system with potential energy *U*(***q***) by starting from the Hamiltonian *H*, which is a function of the 3*N*-dimensional atomic position vector ***q*** and momenta ***p***

1 $$H\left(q,p\right)=\frac{1}{2}p^{T}M{\left(q\right)}^{-1}p+U\left(q\right)$$

The momentum is related to the velocity by ***p*** = *M*(***q***) ***q̇***. The canonical ensemble can be described with the canonical probability density ρ

2 $$\rho \left(q\right)=\frac{\int dpe^{-\beta H}}{\int dqdpe^{-\beta H}}$$

For any operator *A*(***q***) that does not depend on the momenta ***p***, this distribution defines an ensemble average

3 <A>=∫dqρ(q)A(q)

Inserting eq 1 into eq 2, the integration over ***p*** in the nominator can be calculated explicitly

4 $$\int dpe^{-1/2\beta p^{T}M{\left(q\right)}^{-1}p}e^{-\beta U\left(q\right)}=e^{-\beta U\left(q\right)}\sqrt{\frac{{\left(2\pi /\beta \right)}^{3N}}{\det M{\left(q\right)}^{-1}}}$$

If the determinant det *M*(***q***) is enforced to be constant, the term under the square root is canceled by the corresponding term in the denominator of eq 2 and the probability distribution function is thus proportional to the Boltzmann factor *e* ^–β*U*(***q***)^. The freedom to choose *M*(***q***) among the matrix functions with constant determinant can be used to assign higher masses to modes with fast oscillations such as to bond length vibrations.

The equations of motions together with a second-order, symplectic, and time-reversible integrator for the MTMD Hamiltonian were given by Tsuchida^2^ and will not be repeated here. The integrator has, however, essentially the same properties as the integrator we will present in Section 2 for the case of variable cell shape MD. The integrator contains an implicit equation for the propagated mass matrix, but is explicit for the evaluation of the new potential energy. In an initial effort, we have tested an alternative integrator which is explicit for both the mass matrix and the potential energy. This integrator can be derived by directly integrating the velocities with the Lagrangian equations of motion instead of using the canonical momenta, and it is also of second order and time-reversible, but not symplectic. Although the explicit integrator worked well for a duration of a few thousand steps, it was numerically inferior to the symplectic scheme over longer simulation periods and we observed a slow drift to higher energies. A typical plot of the total energy of the explicit MTMD integrator in comparison with the symplectic integrator is shown in Figure 5. The characteristic property of symplectic integrators with fixed time steps is that they are tied to a shadow Hamiltonian, whose energy is an exactly conserved quantity.^3^ For a time-reversible, symplectic integrator, the real Hamiltonian differs from the shadow Hamiltonian by terms of second and higher order in the time step. Therefore, assuming that the time step is small enough that the series converges, the error in the energy conservation is bounded and of second order in the time step. In the Supporting Information, we show a numerical verification of the symplecticness conditions of the integrator.

## 2 Methods

We have generalized MTMD to incorporate the Parrinello–Rahman^4^ method, which is a generalization of MD to variable cell shape systems. The Parrinello–Rahman method is built from a Lagrangian where the dynamical variables are the scaled coordinates {***s***_*i*_}, together with the 3 × 3 matrix ***h***, whose columns are the lattice vectors. The scaled position of the *i*th atom is related to the Cartesian position by ***R***_*i*_ = ***hs***_*i*_. The Lagrangian, after generalizing the method with a position-dependent mass-tensor *M*(***s***, ***h***), reads

5 $$L\left(s,h,ṡ,ḣ\right)=\frac{1}{2}{ṡ}^{T}G\left(s,h\right)ṡ+\frac{1}{2}WTr\left({ḣ}^{T}ḣ\right)-U\left(s,h\right)-P\det h$$

We have introduced *G*(***s***, ***h***)_*ij*_ = ***h*** ^*T*^ *M*(***s***, ***h***)_*ij*_ ***h*** which can be regarded as a mass-weighted metric tensor. The scalar parameter *W* describes an artificial mass of the lattice vectors and *P* is the external pressure. The indices *i*, *j* go over the number of particles *N*. We switch to the Hamiltonian formalism and introduce the canonical momenta

$$p_{s}=\frac{\partial L}{\partial ṡ}=Gṡ$$

6 $$p_{h}=\frac{\partial L}{\partial ḣ}=Wḣ$$

The Hamiltonian is constructed using the Legendre transformation $H=p_{s}^{T}\mathrm{q̇}+Tr\left(p_{h}^{T}ḣ\right)-L$

7 $$H\left(s,h,p_{s},p_{h}\right)=\frac{1}{2}p_{s}^{T}G{\left(s,h\right)}^{-1}p_{s}+\frac{1}{2}\frac{1}{W}Tr\left(p_{h}^{T}p_{h}\right)+U\left(s,h\right)+P\det h$$

From the Hamiltonian, we obtain the following equations of motion

8 ṡ=∂H∂ps=G−1psḣ=∂H∂ph=1Wphṗs,i=−∂H∂s=−12psT∂G−1∂sips−∂U∂siṗh=−∂H∂h=−12psT∂G−1∂hps−∂U∂h−P(det⁡h)h−T

A second-order, time-reversible, symplectic integrator can be constructed with the generalized Störmer-Verlet (leapfrog) algorithm.^3,5^ In the generalized leapfrog algorithm, the partial derivatives of *H* are evaluated at the advanced positions *H*(***q*** ^(*n*)^, ***p*** ^(*n*+1/2)^) for the first half-step and at *H*(***q*** ^(*n*+1)^, ***p*** ^(*n*+1/2)^) for the second half-step. If the Hamiltonian had been separable, *H*(***q***, ***p***) = *T*(***p***) + *V*(***q***), the generalized leapfrog algorithm would have reduced to the standard Störmer-Verlet algorithm, which consists of only explicit equations.

9 $$p_{s,i}^{\left(n+1/2\right)}=p_{s,i}^{\left(n\right)}+\frac{\Delta t}{2}\left[-\frac{1}{2}{p_{s}^{T}}^{\left(n+1/2\right)}{\frac{\partial G^{-1}}{\partial s_{i}}}^{\left(n\right)}p_{s}^{\left(n+1/2\right)}-{\frac{\partial U}{\partial s_{i}}}^{\left(n\right)}\right]$$

$$p_{h}^{\left(n+1/2\right)}=p_{h}^{\left(n\right)}+\frac{\Delta t}{2}\left[-\frac{1}{2}p_{s}^{T\left(n+1/2\right)}{\frac{\partial G^{-1}}{\partial h}}^{\left(n\right)}p_{s}^{\left(n+1/2\right)}-{\frac{\partial U}{\partial h}}^{\left(n\right)}-P\left(\det h^{\left(n\right)}\right){h^{-T}}^{\left(n\right)}\right]$$ 10

11 $$s^{\left(n+1\right)}=s^{\left(n\right)}+\Delta t\left[\frac{1}{2}\left({G^{-1}}^{\left(n\right)}+{G^{-1}}^{\left(n+1\right)}\right)p_{s}^{\left(n+1/2\right)}\right]$$

12 $$h^{\left(n+1\right)}=h^{\left(n\right)}+\Delta t\frac{1}{W}p_{h}^{\left(n+1/2\right)}$$

$$p_{s,i}^{\left(n+1\right)}=p_{s,i}^{\left(n+1/2\right)}+\frac{\Delta t}{2}\left[-\frac{1}{2}p_{s}^{T\left(n+1/2\right)}{\frac{\partial G^{-1}}{\partial s_{i}}}^{\left(n+1\right)}p_{s}^{\left(n+1/2\right)}-{\frac{\partial U}{\partial s_{i}}}^{\left(n+1\right)}\right]$$ 13

$$p_{h}^{\left(n+1\right)}=p_{h}^{\left(n+1/2\right)}+\frac{\Delta t}{2}\left[-\frac{1}{2}p_{s}^{T\left(n+1/2\right)}{\frac{\partial G^{-1}}{\partial h}}^{\left(n+1\right)}p_{s}^{\left(n+1/2\right)}-{\frac{\partial U}{\partial h}}^{\left(n+1\right)}-P\left(\det h^{\left(n+1\right)}\right){h^{-T}}^{\left(n+1\right)}\right]$$ 14

The first eq eq 9 is implicit because ***p***_***s***_^(*n*+1/2)^ appears on the right-hand side. However, the iterative solution is computationally fast because the operators involved are constant. In Section 4, we solved this equation with a simple fixed-point iteration, which required roughly 12 iterations until the update between consecutive iterations fell to close to machine precision. When the time step was increased in Section 3 in order to try the maximal possible time step, sometimes up to 30 iterations were required or the solution did not converge at all, and consequently it was necessary to be content with a reduced time step. Equation 11 is also implicit and more difficult to solve, because the operator *G* ^–1(*n*+1)^ depends on ***s***_*i*_^(*n*+1)^. Hence, during the iterative solution, *G* ^–1^ must be recalculated repeatedly. In Sections 4 and 5, we solved this equation by starting with a single step of a fixed-point iteration followed by a switch to the more efficient Newton–Raphson method. Machine precision was reached after about 4 iterations in total.

The MTMD method is efficient under the condition that the evaluation of the mass matrix is cheap compared to the evaluation of the potential energy. This is likely true if the energy is calculated with a quantum mechanical method and the mass matrix is calculated with a classical method. We will return to this point in Sections 4.3 and 5. The remaining equations are explicit and can be evaluated in the given order, except that eq 12 must be evaluated before eq 11. The expression for the derivative of the *G* matrix is given in the Supporting Information.

## 3 MTMD with Molecular Nitrogen Molecules

To illustrate the mass tensor molecular dynamics technique with a first example, we simulated a molecular nitrogen system, where fictitious masses were assigned to the vibrational and rotational modes of the *N*_2_ molecules. Because of the simple form of the internal vibrations (only a single degree of freedom per molecule), we employed a definition of the mass matrix tailored to diatomic molecules. A definition of the mass matrix for larger molecules will be proposed in Section 4.2.

### 3.1 Mass Matrix for Diatomic Molecules

The standard mass matrix of an *N*-atom system of nitrogen molecules has the form *mI* with *m* the atomic mass of nitrogen and *I* the 3*N*-dimensional identity matrix. In the next step, the masses of certain modes are modified. These modes should be restricted to the motion of single molecules so that, if two modes belong to different molecules, they are orthogonal to each other. The resulting mass matrix has a block diagonal structure with *N*/2 blocks of size 6 × 6. We only need to consider a single block, belonging to one molecule, since all molecules are treated in the same way. Each block can be decomposed into three translational modes, two rotational modes and one vibrational mode

15 $$M_{mol}=m_{trans}\frac{1}{2}\left(\begin{array}{ll} 1 & 1\\ 1 & 1 \end{array}\right)⊗I_{3}+m_{rot}\frac{1}{2}\left(\begin{array}{ll} 1 & -1\\ -1 & 1 \end{array}\right)⊗\left(I_{3}-{\mathrm{d̂}}_{ij}{\mathrm{d̂}}_{ij}^{T}\right)+m_{vib}\frac{1}{2}\left(\begin{array}{ll} 1 & -1\\ -1 & 1 \end{array}\right)⊗{\mathrm{d̂}}_{ij}{\mathrm{d̂}}_{ij}^{T}$$

here, ${\mathrm{d̂}}_{ij}$ is the unit vector pointing from atom *i* to atom *j* and *I*_3_ is the 3-dimensional identity matrix. The symbol ⊗ is the Kronecker product, that multiplies a 2 × 2 matrix and a 3 × 3 matrix to obtain a 6 × 6 matrix. If *m*_trans_ = *m*_rot_ = *m*_vib_ = *m* are all chosen to be the atomic weight of nitrogen, the matrix block reduces to *mI*_6_, which is the standard mass matrix of a nitrogen molecule. The MTMD technique permits assigning different masses to the three kinds of modes.

### 3.2 Computational Details for Nitrogen Gas

The simulations were performed with a simple force field parametrized by Wang et al.,^6^ which for the current purpose has the following potential energy expression

16 $$E_{pot}=\sum_{ijbonded}\frac{1}{2}K_{r}{\left(r_{ij}-r_{0}\right)}^{2}+\sum_{ijnonbonded}\epsilon \left[{\left(\frac{r_{m}}{r_{ij}}\right)}^{12}-2{\left(\frac{r_{m}}{r_{ij}}\right)}^{6}\right]$$

The force field contains four parameters: The equilibrium bond length *r*_0_ = 1.0977 Å, the spring constant *K*_*r*_ = 138.331 eV/Å^2^, the minimum of the Lennard-Jones potential ϵ = 0.003456 eV and the equilibrium interatomic distance *r*_m_ = 3.614 Å.

We have placed 512 nitrogen molecules in a periodic cubic box of initially 32 Å side length, which corresponds approximately to the experimental density of liquid nitrogen. Since the Parinello-Rahman method can give rise to a substantially shearing of the unit cell, we have imposed the constraint that only uniform scaling is allowed. We have implemented this constrained by projecting the rate of change of the lattice vectors, ${ṗ}_{h}$ , to the 1-dimensional subspace of uniform cell scalings before using it to update the lattice vector momenta. The initial lattice vector momenta were chosen to be compatible with this constraint. The kinetic energy was chosen such that the system remained in the liquid phase, but was slightly superheated with a temperature of about 90 K. As a reference case, we performed a standard MD of this system. The simulation was performed in the NpH ensemble, where the energy (more precisely: enthalpy) is a conserved quantity and the external pressure is a parameter, which was set to 0.0001 GPa (atmospheric pressure).

With standard molecular dynamics, the largest stable time step is strongly influenced by the internal vibrational frequency of the nitrogen molecules. We found that the simulation was numerically stable with standard MD up to a time step of 4 fs. We then switched to MTMD, still with the same ensemble and temperature, but with a 100-fold increased vibrational mass and a 4-fold increased rotational mass by modifying *m*_vib_ and *m*_rot_ in eq 15. While the reduction of the vibrational frequency serves the purpose of increasing the possible time step, the reduction of the rotational frequency was done purely for illustrative purposes. With the modified mass matrix, the time step could be increased up to 25 fs. To compare the two methods in the high temperature limit, we also ran both simulations at approximately 1500 K, starting with a cubic cell with initially 480 Å side length, which corresponds to the expected volume in the gas phase. At high temperatures, the maximal stable time step for standard MD was still 4 fs while the maximal stable time step for MTMD dropped to 10 fs. The different behavior of the maximal stable time step with regard to temperature can be explained with the fact that the standard MD time step is limited by the time scale of internal vibrations, which are governed by a harmonic potential, while the MTMD time step is limited by the time scale during molecular collisions, which are governed by the anharmonic Lennard-Jones potential. The Lennard-Jones potential has a rather high curvature whenever two atoms approach more than the hard-sphere diameter of the atoms and this happens mainly at high temperatures.

### 3.3 Results and Discussion for Nitrogen Molecules

A spectral analysis of the atomic motions was performed in liquid nitrogen at 90 K. The motions were filtered to isolate the translational, rotational, and vibrational motions (center-of-mass motions of the molecules, orientation of the molecules relative to a fixed axis, bond length); see Figure 1. With the modified mass in MTMD, the maximum of the vibrational spectrum has decreased from 70.1 ps^–1^ to 7.0 ps^–1^. In addition, the expected shift of the rotational spectrum to roughly half of its previous frequency is visible in the plot. The frequency ranges, where the three modes have most of their weight, have much more overlap after switching to MTMD. The result agrees well with the observed increase of the maximum possible time step. In typical MD applications, the maximal time step is limited to one-10th to one-fifth of the fastest oscillation period. Trying various values of *m*_vib_, we observed that an increase to more than 100 times its standard value did not help improve efficiency, because the translational and rotational parts of the frequency spectrum would dominate in the range of high frequencies. The translational frequencies are mainly originating from the Lennard-Jones part of the force field, which was not considered during the construction of the mass matrix because that would have undermined the block-diagonal structure of the mass matrix. Furthermore, the Lennard-Jones potential is highly anharmonic, and assigning a constant mass to motions parallel to the Lennard-Jones gradient would therefore be ineffective.

**Figure 1:**
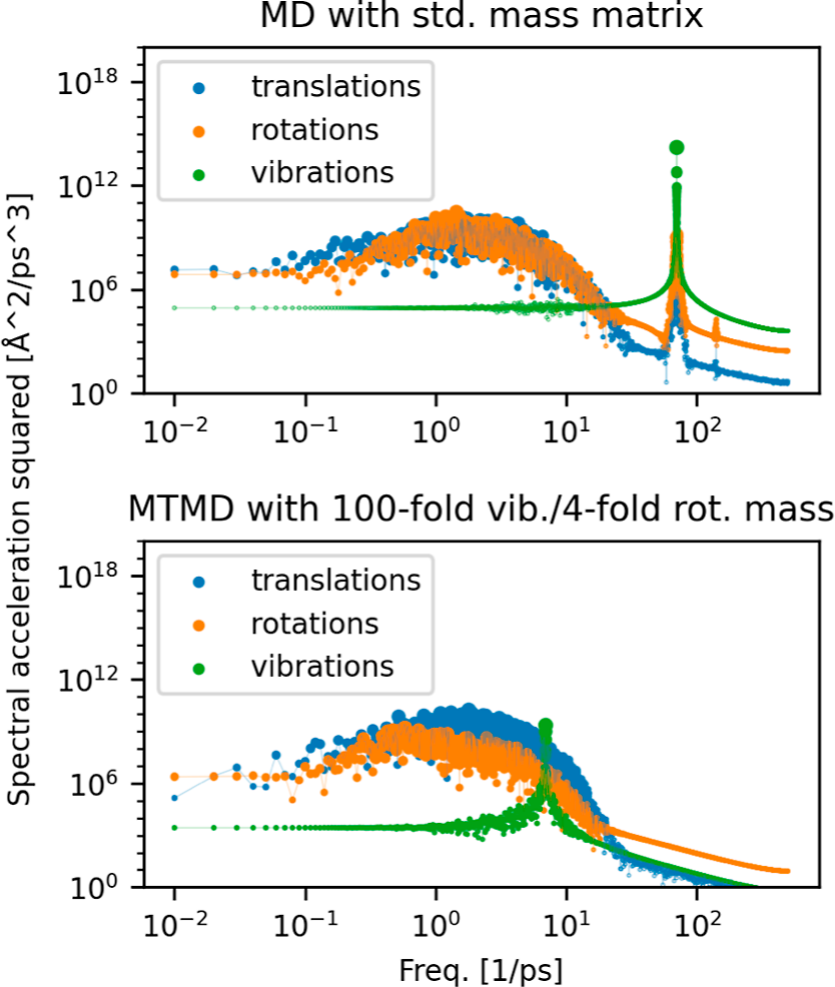
Spectral power of the second derivative of the atomic motions with respect to time, filtered by translational, rotational and vibrational modes. The peak of the vibrational spectrum is downshifted when switching to the modified mass matrix. Also the downshift of the rotational frequencies is vaguely visible.

In the Supporting Information, we present a simulation illustrating the phase transition of molecular nitrogen at its boiling point.

## 4 MTMD for Phase Transitions in Molecular Crystals

### 4.1 Mass Matrix Based on Internal Coordinates

We will now consider systems of molecules that are larger than the diatomic molecules in Section 3 and construct a mass matrix that is suited for molecules with more than one internal degree of freedom. The fastest vibrations in molecular systems are related to covalent bonds, in particular changes in bond lengths (stretching) and to a lesser extent changes in bond angles (bending) and dihedral angles (torsion). A well-constructed mass matrix should aim to slow down the fast vibrational frequencies within a molecule so that the fastest frequencies are of the same order of magnitude as the remaining frequencies. For a purely quadratic potential, all vibrational frequencies could be made equal by choosing a mass matrix that is proportional to the Hessian matrix. For nonharmonic systems, this is not possible, but the Hessian matrix is still a good starting point to construct an improved mass matrix. The Hessian matrix should not be recomputed at each step because Hessian matrices are typically expensive to calculate. Furthermore, even if the Hessian matrix calculation could be afforded, the Hessian matrix can have negative eigenvalues, and a straightforward usage of the Hessian matrix as the mass matrix would break down the MD algorithm. We took the approach of calculating the Hessian matrix only once at the beginning of the MD simulation, assuming that the MD starts from a local minimum of the potential energy surface. The Hessian matrix is then transformed into redundant internal coordinates.^7^ The main assumption is that the Hessian matrix of the initial structure in redundant internal coordinates is a good description of the expected maximal curvatures throughout the MD simulation.

The set of redundant internal coordinates {*q*_*i*_} is constructed by first detecting the covalent bonds. We assume that no bond breaking occurs, and so the set of internal coordinates does not change during the simulation, though it would be interesting to investigate other constructions of the mass matrix, that allow for bond breaking and bond formation or that do not depend on the definition of bonds at all. A bond is detected whenever the interatomic distance between two atoms is less than a critical value that depends on the chemical elements involved. All possible bonds, angles, and dihedral angles that can be constructed from the detected bonds are included in the list of internal coordinates.

The first derivatives of a set of internal coordinates {*q*_*i*_} consisting of bond lengths, angles and dihedral angles as a function of 3*N* Cartesian coordinates {*x*_*j*_} is called Wilson’s B matrix

17 $$B_{ij}=\frac{\partial q_{i}}{\partial x_{j}}$$

The Wilson *B* matrix is a rectangular matrix with often more rows than columns (thus the name redundant). It allows to transform the gradient in Cartesian coordinates ***g***_*x*_ to internal coordinates ***g***_*q*_ (forward transformation) and vice versa (backward transformation)

18 gq=BT+gxgx=BTgq

The forward transformation is done with the pseudoinverse *B* ^+^ which is a generalization of the standard inverse and exists for any rectangular matrix. When *B* ^+^ is calculated numerically, a threshold must be specified below which singular values are treated as zero. We used 10^–2^ times the largest singular value as a threshold. Angles were measured in radians, so that the bond lengths and angles were of similar magnitude. The transformation rules for Hessian matrices are

19 $$H_{q}=B^{T+}\left(H_{x}-K\right)B^{+}$$

20 $$H_{x}=B^{T}H_{q}B+K$$

The matrix $K_{jk}=\underset{i}{\sum }g_{q,i}\frac{\partial^{2}q_{i}}{\partial x_{j}\partial x_{k}}$ is the sum of the second derivatives of the internal coordinates, weighted by the gradient along that particular direction in the internal coordinates. We do not need the *K*-matrix to transform the Hessian matrix to internal coordinates because we apply the forward transformation only at the initial local minimum, where the gradient and the *K*-matrix vanish

21 $$H_{q,0}=B_{0}^{T+}H_{x,0}B_{0}^{+}$$

The matrix *H*_*q*,0_ is positive semidefinite since it is the Hessian matrix of a local minimum. In addition, it is block diagonal, with each molecule building a separate block. Because the initial Hessian matrix *H*_*x*,0_ is calculated for a molecule embedded in a crystal, its projection to the internal coordinate system of a molecule is not equal to the Hessian matrix of a free molecule. In particular, very soft modes that involve large relative motions of distant parts of the molecule are suppressed. The actual Hessian matrices *H*_*q*_(*t*) that occur along the MD trajectory at times *t* differ from the initial Hessian matrix in internal coordinates and are generally indefinite. But, likely, the curvatures in the same directions of the internal coordinates either remain of a similar magnitude or become negative, e.g. when the position is shifted from a local minimum on the PES to a saddle point. For the current purpose, it is only important that the curvatures and therefore the vibrational frequencies are not significantly underestimated. To construct the mass matrix, the initial Hessian matrix in internal coordinates is converted back to Cartesian coordinates, this time using the Wilson *B* matrix of the actual position instead of the one used during the forward transformation. In principle, the backward transformation using formula eq 20 would involve the *K*-matrix, but this would be problematic because, first, the *K*-matrix contains the second derivatives of the internal coordinates, and treatment in mass tensor molecular dynamics would then require third derivatives, and second, more severely, the *K*-matrix might introduce negative curvatures in the back-transformed Hessian matrix, since the *K*-matrix is indefinite. Hence, it is reasonable to omit the *K*-matrix during the backward transformation. This amounts to the assumption that we treat not only the Hessian matrix in internal coordinates to be constant but also the gradient to be zero. Without the *K*-matrix term, the back-transformed Hessian matrix becomes positive semidefinite.

The estimated curvatures in the directions of translations and rotations of whole molecules are still zero when the back-transformed Hessian matrix is used, and the curvatures of other modes might also be zero if there are too few internal coordinates to uniquely describe a molecule. Zero-curvature modes are not allowed in the mass matrix, since relative motions between different molecules need to have kinetic energy, and thereby inertia. To correct for this, we add the positive definite physical mass matrix *M*_*p*_ to the mass matrix. Finally, a positive definite mass matrix is constructed as follows

22 $$M\left(q\right)=\left(wB^{T}\left(q\right)H_{q,0}B\left(q\right)+M_{p}\right)\times c\left(q\right)$$

Here, ***q*** is used for the generalized coordinates as in Section 1 and is not related to the internal coordinates {*q*_*i*_} from the previous discussion. The summation of the two terms in eq 22 is controlled by specifying a weight factor *w* for the Hessian matrix term. Although the Hessian matrix in internal coordinates *H*_*q*,0_ is approximated by a constant matrix, the mass matrix *M* is a function of the positions ***q*** through the Wilson *B*-matrix. In addition, the overall factor *c* that rescales the mass matrix so that the determinant stays constant during the simulation also depends on the positions. The initial value *c*(***q***_0_) is still arbitrary. Tsuchida chose it so that the fastest vibrational frequencies in the modified and unmodified calculations coincide. We use a mathematically equivalent approach, which, however, changes the physical interpretation of the simulation time scale. We set *c*(***q***_0_) = 1, which means that the Hessian term of the mass matrix is basically just added without rescaling the physical term. Still, *c*(***q***) fluctuates slightly around 1 because the determinant must be kept constant, but this fluctuation is only on the order of one percent. We think that adding the Hessian term without rescaling the physical term is justified because the idea of MTMD is to slow down unimportant modes, while the important, structurally changing modes are ideally not affected. This means in particular that translations of molecules, and hopefully also other important reaction modes, should happen on the same time scales as in the unmodified physical case.

The presented construction of the mass matrix resembles the first variant of the MTMD mass matrix described by Tsuchida (eq 10 in ref 2), but it extends his definition by including dihedral angles and containing coupling terms between different internal coordinates. Tsuchida finally arrives at a second variant in which a filtering function is applied to the eigenvalues of the Hessian matrix to make them positive. We do not consider this variant here because our construction of the mass matrix is already positive definite through the omission of the K-matrix.

### 4.2 Computational Details for Molecular Crystals

To demonstrate MTMD in a variable cell shape system, we studied the transition of *N*-(4-Methylbenzylidene)-4-methylaniline from polymorph II to polymorph III, see Figure 2. Both polymorphs have been observed experimentally.^8,9^ In a recent study by our group,^10^ this molecule was chosen as a model system for molecular crystals that exhibit polymorphism. The referred study shows, based on DFT calculations, that polymorph III has a lower ground-state energy, but polymorph II is stabilized at room temperature due to structural tolerance. We modeled this system with variable cell shape at the minimum size of 62 atoms (two molecules). A good order parameter to distinguish the two polymorphs is the angle between the main axes of the two molecules. The main axis is defined as the direction between the center of masses of the two carbon rings in each molecule. This angle is about 0° for polymorph II and about 90° for polymorph III. The transition from polymorph II to polymorph III at room temperature is a rare event and cannot be observed with a straightforward MD simulation in a reasonable time.^10^ This situation could in principle be addressed by enhanced sampling methods, which simulate rare events using bias forces.^11,12^ We instead used a more physical approach and increased the temperature to about 750 K to overcome the energy barrier and facilitate the transition. Further we replaced DFT with DFTB+^13^ with the mio-1–1 DFTB parameter set.^14^ A single *k*-point was used and D4 dispersion correction^15^ was added. Switching to DFTB+ accelerated the calculation by 2 orders of magnitude. Unlike many force fields, DFTB + allows bond breaking and bond formation,^16^ although bond breaking is not involved in the transition we examined. DFTB+ with dispersion correction has also been used in another study of molecular crystals.^17^ A compensatory measure to prevent the system from evaporating after increasing the temperature was to increase the external pressure to 1 GPa. Although the modified temperature and pressure influence the ensemble that is sampled, the results are still useful to gain qualitative insight into the reaction mechanisms involved and into the ordering of free energies of different phases. Quantitative results can, in principle, be obtained by postprocessing the reaction paths with calculations at ambient temperature and pressure;^18^ however, for the current study, we were only interested in optimizing the MD efficiency. We thus calculated the transition rate at increased temperature and pressure, making a comparison between standard MD and MTMD.

**Figure 2:**
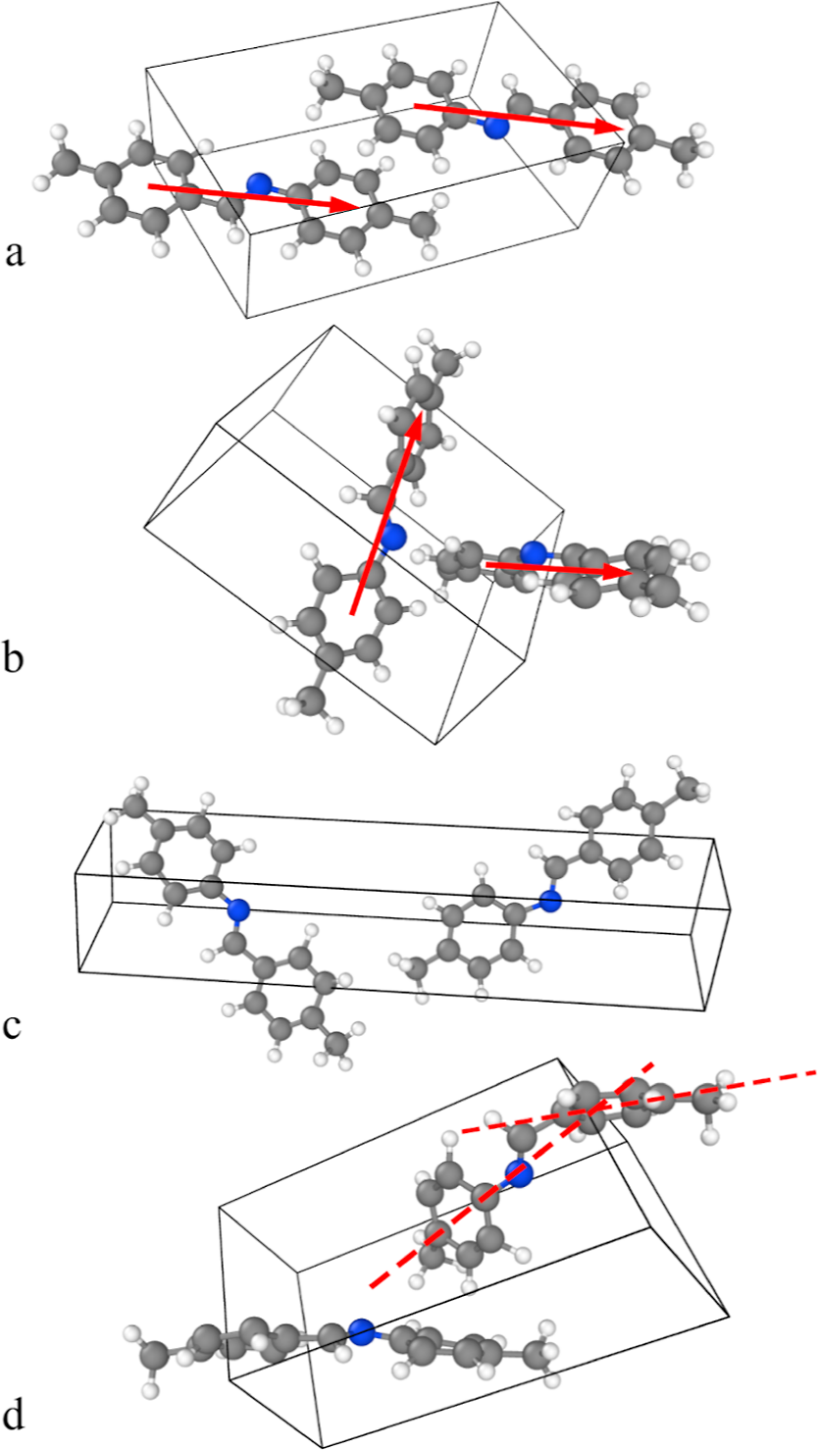
(a) *N*-(4-Methylbenzylidene)-4-methylaniline in the polymorph II phase. The main axes of the two molecules (red arrows) are parallel. (b) Polymorph III. The main axes of the two molecules (red arrows) are nearly orthogonal. (c) Finite temperature MD sample in a nonstandard form, where the main axes are nearly orthogonal as in polymorph III but the unit cell is much more elongated than in the reference structure. (d) Finite temperature MD sample in a nonstandard form, where the main axes are nearly orthogonal as in polymorph III, but one of the molecules has a considerable kink (the angle between the two dashed axes through the two carbon rings of the same molecule is larger than 30°).

To better assess the advantage of MTMD, simulations with the simple mass repartitioning technique were also included in the comparison, where the mass matrix was implemented as proportional to the identity matrix and unchanged total mass, $M_{p}=\frac{1}{N}\left(\sum_{i=1}^{N}m_{i}\right)I_{3N}$ . This choice of *M*_*p*_ was also used for MTMD when implementing eq 22.

To determine the optimal MD time step and weight parameter, we first assessed the reduction of the highest vibrational frequencies by calculating the Hessian matrices and mass matrices for a data set consisting of the ground state of polymorph II and of 100 configurations from a preliminary MD run at 750 K in the polymorph II phase. We compared six different choices of the mass matrix: The standard physical mass matrix, mass repartitioning with a constant mass matrix, and four variants of MTMD mass matrices. The MTMD mass matrices were constructed using the assumption of an approximately constant Hessian matrix in internal coordinates. Three different sets of internal coordinates of increasing size were compared. The first set contained bond lengths only, the second set bond lengths and bond angles, and the last set additionally dihedral angles. In one variant, the off-diagonal elements in the internal coordinates Hessian matrix were artificially set to zero, since this is closest to the definition used by Tsuchida.^2^ The vibrational spectrum was obtained from the generalized eigenvalues ω_*j*_^2^ of *H* and *M* which are identical to the eigenvalues of the mass-weighted Hessian matrix *M* ^–1/2^ *HM* ^–1/2^. The highest frequency is max ω_*j*_/2π. The average of the highest frequencies of the finite-temperature samples as well as the highest frequency of the ground state of polymorph II are plotted in Figure 3. The results show a systematic reduction of the highest vibrational frequencies when going to more sophisticated constructions of the mass matrix. The best reduction is obtained with a mass matrix that is based on the full set of redundant internal coordinates and includes off-diagonal elements. This mass matrix was therefore used in all further MTMD calculations. The inclusion of off-diagonal elements does not make a large difference, however, which is understandable since the internal coordinates were invented exactly for the purpose of reducing the coupling between different coordinates.

**Figure 3:**
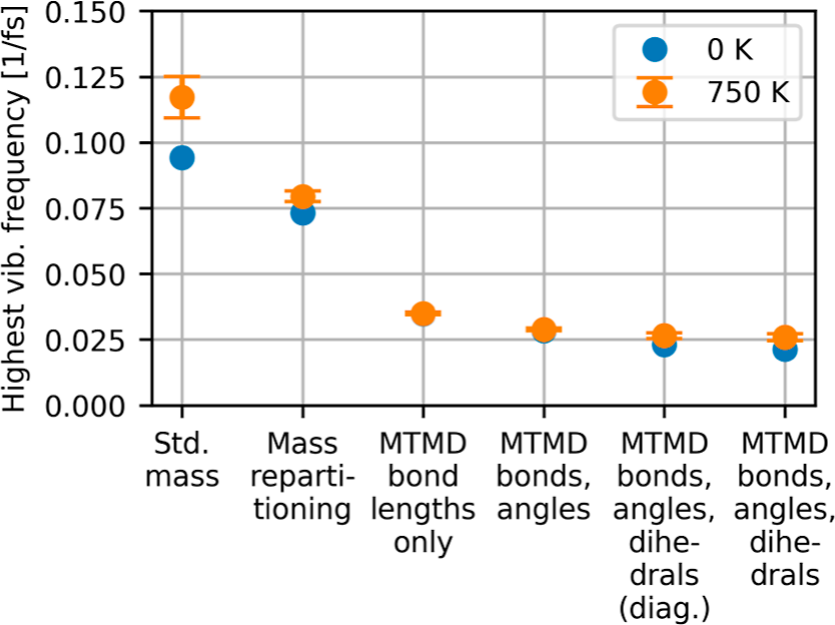
Orange markers show the average of the highest vibrational frequencies and standard deviations of a data set of configurations obtained from an MD run at 750 K. Six different definitions of the mass matrix are compared. The blue markers show the highest vibrational frequency at the geometry optimized polymorph II structure.

We empirically chose a weight factor of *w* = 0.5 u·Å^2^/eV because higher values led to an increased failure rate of MTMD. The maximum of the vibrational spectrum is shown in Figure 4 as a function of *w*. Although the maximum frequency of the ground state can be further decreased with ever higher values of *w*, the maximum frequency over the finite temperature data set shows saturation and cannot be further reduced with weight value *w* > 1.0.

**Figure 4:**
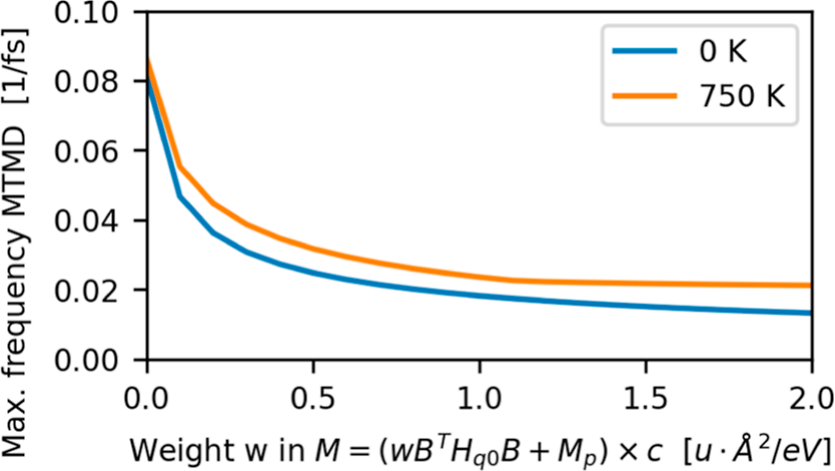
Highest frequencies of the mass-weighted Hessian matrix of the molecular crystals as a function of the weight *w*. The blue curve shows the highest frequency of the groundstate of polymorph II, while the orange curve shows the maximum from the highest frequencies in the finite temperature data set.

The time step was chosen separately for each method (standard MD, mass repartitioning, MTMD) to be exactly one-tenth of the fastest vibrational oscillation period of the mass-weighted Hessian matrix of the initial structure. To parallelize the calculations, we started 2000 independent runs for each method with different random initial velocities taken from a Boltzmann distribution at 1500 K. Each run was carried out for a total of 20′000 time steps. The MD simulation was performed in the microcanonical ensemble because it allowed us to easily check energy conservation. The calculations were repeated with a time step of 1/20 of the fastest vibrational period instead of 1/10 to reduce any error that might be attributed to the choice of time step. The calculations with smaller time step were carried out over 40′000 steps per run in order to acquire the same simulation time.

### 4.3 Results and Discussion on Molecular Crystals

The main result of our calculation was a comparison between standard MD and MTMD regarding the efficiency of simulating the transition from polymorph II to polymorph III. Since all the runs started at a local minimum, the temperature dropped from the initial value of 1500 K and equilibrated rapidly at around 750 K. After the initial equilibration phase, the total energy fluctuated within a range of about 0.2 eV in both MTMD and regular MD with no noticeable drift, which is a characteristic property of a symplectic integrator. A typical plot of the total energy evolution of an MTMD is shown in Figure 5. For comparison, an MTMD run with an alternative nonsymplectic integrator is shown as well. The nonsymplectic integrator exhibited a slow but relevant drift of the total energy.

**Figure 5:**
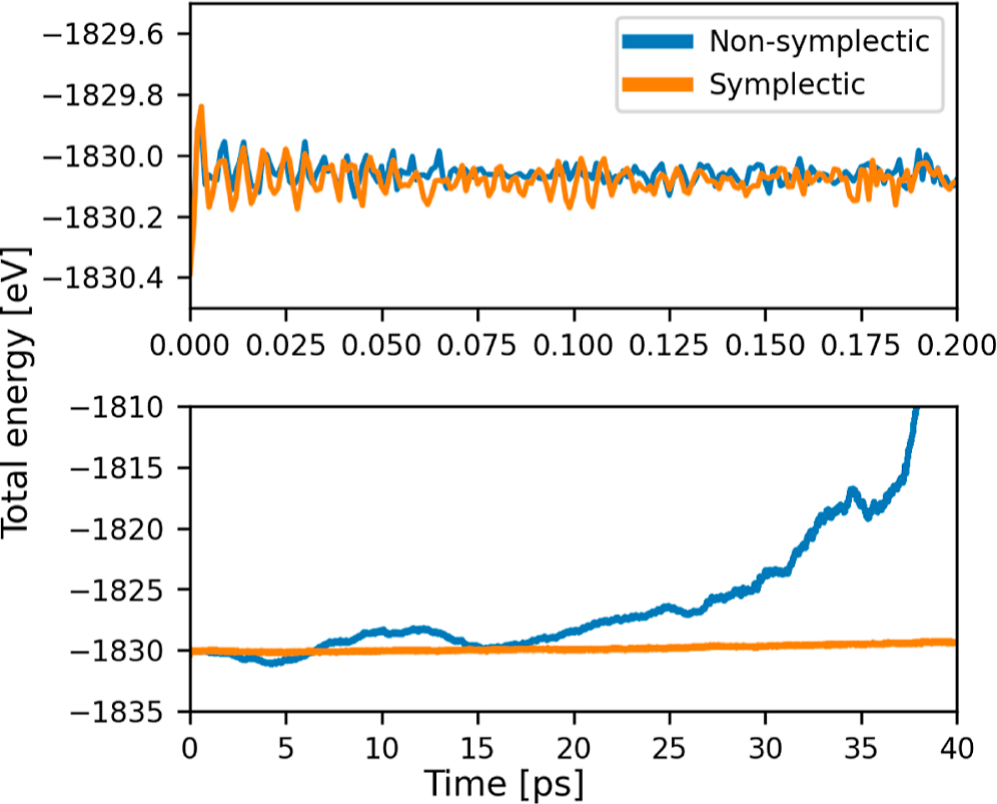
To check energy conservation, the total energy of MTMD is plotted for a typical run of both a nonsymplectic and a symplectic algorithm. The upper subplot shows a zoomed view of the energy at the beginning of the trajectory, while the second subplot shows the energy along the whole trajectory. The short-term error is very similar for both methods, while the symplectic algorithm has a much better long-term energy conservation.

Most runs were stable during the planned simulation time. Only in 30 out of 2000 MTMD runs did the total energy exhibit a sudden jump or the integration failed. The regular MD ran without incident. We have looked closer at the trajectories of three out of the failed runs, and we noticed that in all cases long before the energy jump, the shape of the simulation cell became extremely distorted and flat, which is something that we have repeatedly seen in Parrinello–Rahman dynamics at higher temperatures. Shortly before the energy jump, one of the methyl groups lost its regular shape and became distorted, and then one hydrogen atom detached from the methyl group. So, the root cause of the integration failure is the violation of the no-bond-breaking assumption. However, we note that these failures occurred in fewer than one instance per million integration steps and could easily be handled by restarting the simulation.

The transition rate from polymorph II to polymorph III was measured by automatically detecting occurrences of a nearly orthogonal orientation of the two molecules. This event was defined as the increase of the intermolecular angle to above 80° in a rolling average over 100 time steps. In some cases, the angle criterion was met, but the cell shape did not match the expected cell shape of the polymorph III phase but was, e.g., much more elongated than the cell of the reference polymorph III structure, or one of the two molecules had a considerable intramolecular kink of more than 30° (defined by the angle between the two axes through the two carbon rings of the same molecule, see Figure 2). These cases were not included in the count of transition events. The transition rates for each method are shown in Table 1. The calculations with smaller time steps have shown qualitatively similar results, although the transition rates were consistently lower. We suspect that a higher time step effectively reduces the activation energy and thereby increases the transition rate, because the transition rate is an exponential function of the activation energy. It is known that the choice of masses and the choice of the MD time step can influence reaction rates; in particular, in one study, the protein ligand binding rate was reduced when switching to mass repartitioning and increasing the time step.^19^ Because the time-step-dependent energy error scales with the square of the time step and because the ratio between transition rates obtained with small and large time steps remained similar when switching between MD and MTMD, we conclude that the reaction rate is not largely affected by the switch from MD to MTMD.

**Table 1:** Counts of Observed Transitions from Polymorph II to Polymorph III as a Total of all Runs^a^

method	time step [fs]	simul time [ns]	transit count	transit rate [ns^–1^]
std. MD	1.061	42.44	5	0.118
mass rew	1.365	54.60	5	0.092
MTMD	4.685	187.40	18	0.096
std. MD	0.530	42.44	2	0.047
mass rew	0.682	54.60	0	0.000
MTMD	2.343	187.40	12	0.064

a Each method (standard MD, mass reweighting MD, MTMD) was performed with 2′000 independent runs and 20′000 time steps per run. The runs were repeated with half the step size and twice as many steps. MTMD allows for a much longer simulation time with the same number of steps. The observed transition rates were similar for all methods, which confirms the efficiency gain with MTMD.

In summary, the MTMD method in this molecular crystal system allowed us to increase the time step by a factor of 4.4 and thus sample more transition events per time step by the same factor. In this paper, we measured performance in terms of the number of force evaluations. A speed-up in wall-clock time was not clearly achieved with the current setting, because a quantum-mechanical force calculation with DFTB+ took on average only 0.42 s, while the computational overhead associated with the calculation of the mass matrix was 1.21 s. This ratio would certainly have changed if we had switched to the DFT level. We consider the current application merely as a proof-of-concept for the MTMD method, with DFTB+ serving as a placeholder for a more demanding calculation. The single most time-consuming part that contributed to the MTMD overhead was the analytical calculation of the derivative of the Wilson B matrix during the iterative recalculation of the masstensor while solving eq 11. Matrix inversions and matrix multiplications in the same loop also played a role, but were less critical. The scaling of the computational overhead of MTMD is linear with the system size, as long as the size of the molecules remains constant, and therefore the size of the blocks in the block-diagonal mass matrix are bounded.

## 5 MTMD of Liquid Water

Liquid water was chosen by Tsuchida as a benchmark application in his original study of ab initio mass tensor molecular dynamics,^2^ because the covalent bonding pattern in the water molecules remains constant during the simulation and can be addressed well with mass tensor molecular dynamics. The remaining interactions, such as hydrogen bonds and van der Waals interactions, are complex enough, so that the simulation benefits from a first principle treatment. In this section, we study the adaption of the MTMD simulation of liquid water to the isobaric-isoenthalpic (NpH) ensemble. The simulation of liquid water with ab initio molecular dynamics has been a long-standing problem in physical chemistry. We refer to a recent article for a summary of the topic.^20^

### 5.1 Mass Matrix for Water Molecules

The mass matrix was constructed with the same procedure as for the molecular crystals in Section 4.1. A reference structure of 64 water molecules was prepared in the cubic ice (Ic) phase, in which all molecules are equivalent. This structure was relaxed on the DFT potential energy surface and the Hessian matrix in redundant internal coordinates was calculated with finite differences. The internal coordinates consist of only bond lengths and bond angles. The Hessian matrix of cubic ice in redundant internal coordinates served as an approximation to the Hessian matrix of liquid water in redundant internal coordinates. The positive semidefinite approximation of Hessian matrix in Cartesian coordinates was obtained by using the back-transformation formula of eq 21. A positive definite mass matrix was constructed by multiplying the Hessian matrix with a weight factor of 0.2 u·Å^2^/eV and adding the physical masses of the atoms. The choice of the weight factor was guided by the observation, that the reduction of the maximal frequency saturated when even higher values were used. Unlike in Section 4.1, no mass-repartitioning was used, because we were interested in the dynamical property of water self-diffusion, which is a process that, in addition to translations of molecules, consists mainly of breaking and reformation of hydrogen bonds, and this mechanism would have been retarded by mass repartitioning.

### 5.2 Computational Details for Liquid Water

DFT calculations were performed using the CP2K quantum chemistry software.^21^ We followed Miceli et al.^22^ for the choice of functional by combining the (semi)­local PBE functional with the nonlocal rVV10-b9.3 functional, where the parameter *b* determines the short-range damping of the dispersion interaction, and a value of *b* = 9.3 was found to provide the optimal fit to the experimental density of liquid water,^22^ while an increased density is observed with the default value of *b* = 6.3.^23^ Following Miceli et al.,^24^ the TZV2P basis set was used as the primary basis and a plane wave basis as the auxiliary basis with a cutoff energy of 800 Ry. A high plane wave cutoff is necessary to reduce the influence of discontinuities in the number of grid points when the size of the unit cell changes.^25^ The core electrons are represented by a Goedecker-Teter-Hutter pseudopotential.^26^ Following Miceli et al.,^24^ liquid water was modeled with 64 water molecules in a cubic box. Molecular dynamics was performed on the Born–Oppenheimer surface in the isobaric-isoenthalpic ensemble (NpH). A Parrinello–Rahman barostat was applied with an external pressure of 0.0001 GPa and a lattice vector mass of 20 au. A constraint was imposed to retain the initial cubic symmetry of the cell.

Because the cubic ice structure did not spontaneously melt in MD, we prepared an additional structure as the initial state of liquid water. This structure was extracted from a force-field molecular dynamics simulation with 64 water molecules in a cubic cell at the experimental density of liquid water. The simulation was then continued on the DFT level and the total energy was manually adjusted by reducing the kinetic energy after every few MD steps until the resulting temperature was within the experimental temperature range of liquid water.

A time step of one-tenth of the fastest oscillation mode was chosen. This corresponds to a time step of 2.89 fs for MTMD and 1.02 fs for standard MD. The time step for standard MD is about twice as large as in many other DFT MD simulations of liquid water,^2,24^ because the main focus of this work is on examining the behavior with large time steps rather than achieving highly accurate physical results. The relative enlargement of the time step when switching from standard MD to MTMD was 2.8, which is slightly lower than the efficiency gain of 3.5 reported by Tsuchida.^2^ This can perhaps be attributed to a different functional or to the increased density in our simulation and, thereby, to an increased strength of the hydrogen bonds. To avoid an unwanted drift of the total energy in the MTMD simulation, we increased the default convergence threshold of the self-consistency cycle in CP2K by 2 orders of magnitude (EPS_SCF = 1 × 10^–7^) and chose a time-reversible wave function guess method, which are both well-known measures to reduce a systematic energy drift.^27^ Large time steps, as used in MTMD, also contribute to the drift, in our experience. The additional computational costs of these measures could have been avoided if a thermostat had been used to compensate for the energy drift.

The simulations were conducted at 350 K or slightly more to ensure liquid-like behavior of the simulated water. This is a common practice,^2,22,24,28^ because at room temperature the self-diffusion coefficient can intermittently become very low and does not converge. Simulations were conducted over 10 ps with standard MD and 17 ps with MTMD, corresponding to 9815 and 5993 steps, respectively. The wall-clock time for a single-point DFT calculation with CP2K, distributed over 2 Nvidia Grace Hopper GH200 nodes, was 34 s, whereas the overhead related to the mass matrix calculation in MTMD was 2 s without any parallelization.

### 5.3 Results and Discussion on the Liquid Water Simulation

The total energy remained constant with a maximum deviation of 1 meV/atom in both methods standard MD and MTMD. The temperature and volume showed fluctuations as expected for a sampling of the NpH ensemble; see Figure 6. Both simulations did not completely converge, as can be recognized in the plots by the presence of small low-frequency fluctuations. This behavior is probably an artifact of the finite size of the system. All expectation values were calculated after skipping an initial equilibration period of 1 ps. The average temperature was 378 and 379 K for the standard MD and MTMD runs, respectively. The density was 946 kg/m^3^ for standard MD and 922 kg/m^3^ for MTMD, both slightly below the value of 990 kg/m^3^ reported by Miceli et al., who used the same functional but different basis set.^22^ The density is also below the experimental value.

**Figure 6:**
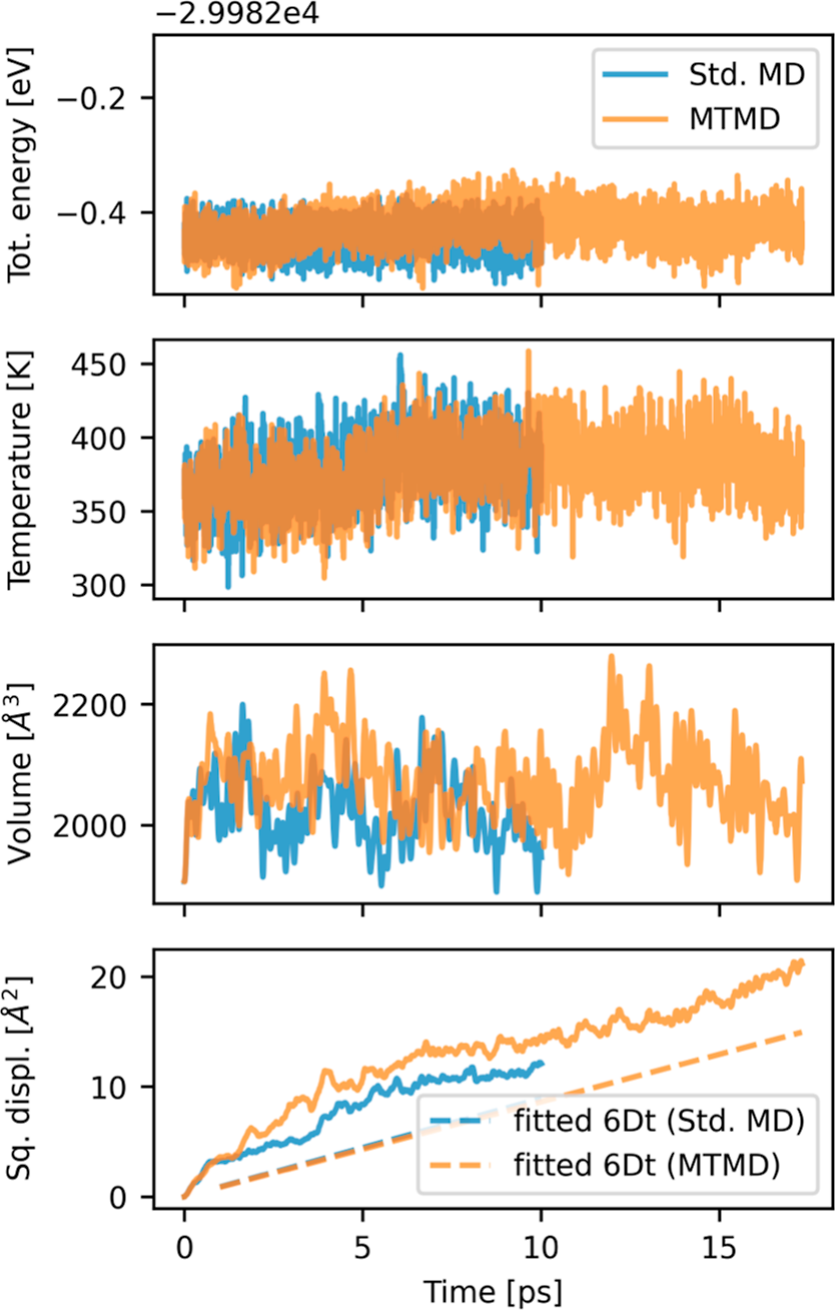
Total energy, instantaneous temperature, volume and squared displacement per oxygen atom in the simulations of liquid water with either standard MD or MTMD. The dashed lines show the fits for the diffusion coefficient *D*.

The trajectories of the standard MD and MTMD simulations of liquid water were used to compute the radial distribution function (oxygen–oxygen distance); see Figure 7. The location of the first peak is at 2.74 Å for both methods with a height of 2.83 for standard MD and 2.87 for MTMD, respectively. The two simulations are in very good agreement with each other and also agree with calculations by Miceli et al.^22,24^ For reference, we also show the experimental radial distribution function reported by Skinner et al.,^29^ which was measured under ambient conditions. Compared with the experiment, the DFT calculations result in a considerable overstructuring of the liquid water, which is also noticeable by the increased volume.

**Figure 7:**
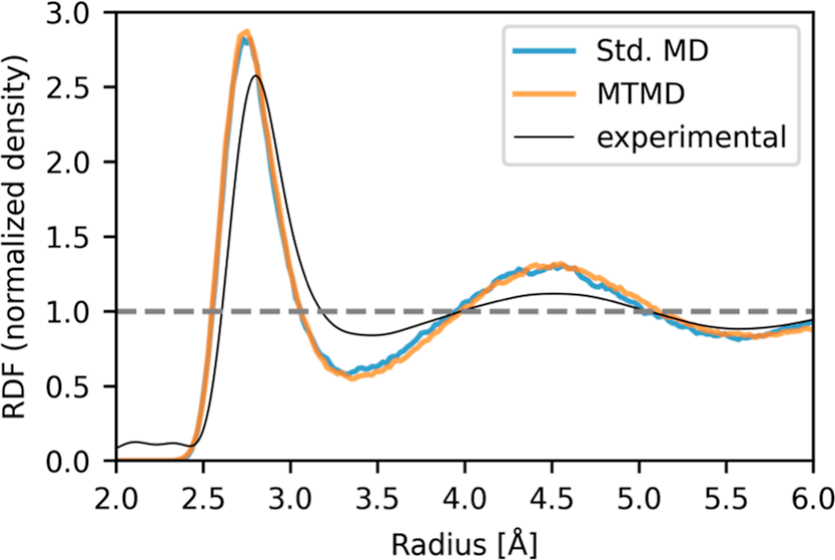
Radial distribution function (RDF) during the liquid water simulation with standard MD and MTMD. The black line shows the experimental result.

There is no rigorous theoretical reason to assume that the dynamical properties in molecular dynamics remain unchanged when the mass matrix is modified. However, not all dynamical properties are affected to the same extent. While the intramolecular vibrational frequencies are predictably affected, as discussed in Section 3, we suppose that dynamical observables at scales larger than single molecules remain more or less unchanged. In this section, we discuss the self-diffusion coefficient of water molecules. The squared displacements of the oxygen atoms, caused by self-diffusion, are plotted in Figure 6.

The self-diffusion coefficient *D* was calculated from the trajectories of the oxygen atoms in both methods with the help of the ASE library,^30^ which generates a fit using the formula ⟨| ***R*** (*t*)-***R*** (0)|^2^⟩ = 6*Dt* + intercept. The fitted self-diffusion coefficients were found to be 0.15 ± 0.02 Å^2^/ps and 0.14 ± 0.02 Å^2^/ps, respectively, for standard MD and MTMD at the simulated temperatures. This shows a very good agreement of this dynamical property calculated with MTMD as compared with the reference calculation. The experimental diffusion coefficient is 0.23 Å^2^/ps under ambient conditions.^31^ The deviation from the experiment reflects an overestimation of the binding strength of the hydrogen bonds by the DFT functional.

In summary, the usage of MTMD for the simulation of liquid-water speeds up the calculations by up to a factor of 2.8. A part of this benefit is diminished because the mass matrix computation induces a small overhead and because of the necessity to have a tighter convergence of the DFT calculation to avoid a systematic energy drift.

## 6 Conclusions

Modifications of the standard Hamiltonian have been successfully used to solve specific tasks such as controlling the pressure and temperature in molecular dynamics. Most of these modifications change the degrees of freedom of the system. The MTMD method, on the other hand, leaves the degrees of freedom of the system unchanged but takes advantage of the freedom to redefine the mass matrix as a position-dependent matrix-valued function. With careful choice of the mass matrix, the dynamics of the system can be substantially modified with the goal of increasing the sampling efficiency without changing the equilibrium properties. The current study has shown that, beyond calculating equilibrium properties, MTMD can also answer dynamical questions such as the calculation of reaction rates or diffusion coefficients. The correspondence between the reaction rates computed with the modified and unmodified mass matrix is not rigorous but valid under the assumption that the slowed-down motions, like local vibrations, and the large-scale motions resulting in relevant transitions do not interfere, which has been demonstrated numerically. The method could be combined in a straightforward way with Parrinello–Rahman dynamics to study transitions in crystalline systems or to simulate liquids. In numerical experiments, an efficiency gain in terms of the number of necessary steps to observe a comparable evolution of the system by a factor of 2.8 to 4.4 were observed compared to standard MD.

The proposed integrator contains an iterative computation of the mass matrix such that several (typically four) recalculations of the mass matrix per time step are necessary. The integrator is semiexplicit in the sense that only one force evaluation per time step is necessary.^32^ Optionally, an explicit but nonsymplectic integrator can be used instead.

While we have focused on systems consisting of molecules with constant bonding patterns and on a mass matrix redefinition that only affects internal vibrations of molecules, a more widely applicable definition of the mass matrix would deserve further investigation. A crucial point for the construction of a mass matrix is that modes with fast oscillations, such as bond length stretching, can be identified in a well-defined and smooth way. In the current paper, we have focused on the special case where the mass matrix is block diagonal with one block per molecule and where the molecules are small or moderately sized. In this setting, the scaling of the MTMD method is linear. Still, because of the computational overhead associated with the MTMD method, a speed-up in wall-clock time is only achievable if the bottleneck of the simulation is a quantum mechanical energy and force calculation.

A reference implementation of the MTMD method in Python is available as an open-source library at https://gitlab.com/goedeckergroup/masstensormoleculardynamics.

## Supplementary Material



## Notes

Numerical proof of the symplecticness. Simulating phase transition at the boiling point of liquid nitrogen. Derivatives of the mass matrix (PDF)

## References

[1] Bennett C. H. (1975). Mass tensor
molecular dynamics. J. Comput. Phys..

[2] Tsuchida E. (2011). Ab initio
mass tensor molecular dynamics. J. Chem. Phys..

[3] Leimkuhler, B. ; Reich, S. Simulating Hamiltonian Dynamics; Cambridge Monographs on Applied and Computational Mathematics; Cambridge University Press, 2005, pp 70–104.

[4] Parrinello M., Rahman A. (1980). Crystal Structure and Pair Potentials:
A Molecular-Dynamics
Study. Phys. Rev. Lett..

[5] Melchionna S. (2006). Numerical
integration of projective Hamiltonian dynamics. Mol. Phys..

[6] Wang S., Hou K., Heinz H. (2021). Accurate and
Compatible Force Fields for Molecular
Oxygen, Nitrogen, and Hydrogen to Simulate Gases, Electrolytes, and
Heterogeneous Interfaces. J. Chem. Theory Comput..

[7] Bakken V., Helgaker T. (2002). The efficient optimization
of molecular geometries
using redundant internal coordinates. J. Chem.
Phys..

[8] Bernstein J., Bar I., Christensen A. (1976). Molecular
conformation and electronic structure. IV. *p*-(*N*-Methylbenzylidene)-*p*-methylaniline (form
III). Acta Crystallogr.,
Sect. B.

[9] Bar I., Bernstein J. (1977). Molecular
conformation and electronic structure. V.
The crystal and molecular structure of *N*-(*p*-methylbenzylidine)-*p*-methylaniline (form
II). Acta Crystallogr., Sect. B.

[10] Krummenacher, M. ; Sommer-Jörgensen, M. ; Gubler, M. ; Finkler, J. A. ; Khajehpasha, E. R. ; Fisicaro, G. ; Goedecker, S. Implications of the multi-minima character of molecular crystal phases onto the free energy. 2025, https://arxiv.org/abs/2501.18372, accessed (Jan 30, 2025).

[11] Tiwary P., Parrinello M. (2013). From Metadynamics
to Dynamics. Phys. Rev. Lett..

[12] Ray D., Parrinello M. (2023). Kinetics from
Metadynamics: Principles, Applications,
and Outlook. J. Chem. Theory Comput..

[13] Hourahine B., Aradi B., Blum V., Bonafé F., Buccheri A., Camacho C., Cevallos C., Deshaye M. Y., Dumitrică T., Dominguez A., Ehlert S., Elstner M., van der Heide T., Hermann J., Irle S., Kranz J. J., Köhler C., Kowalczyk T., Kubař T., Lee I. S., Lutsker V., Maurer R. J., Min S. K., Mitchell I., Negre C., Niehaus T. A., Niklasson A. M. N., Page A. J., Pecchia A., Penazzi G., Persson M. P., Řezáč J., Sánchez C. G., Sternberg M., Stöhr M., Stuckenberg F., Tkatchenko A., Yu V. W.-z., Frauenheim T. (2020). DFTB+, a software
package for efficient approximate density functional theory based
atomistic simulations. J. Chem. Phys..

[14] Elstner M., Porezag D., Jungnickel G., Elsner J., Haugk M., Frauenheim T., Suhai S., Seifert G. (1998). Self-consistent-charge
density-functional tight-binding method for simulations of complex
materials properties. Phys. Rev. B.

[15] Caldeweyher E., Ehlert S., Hansen A., Neugebauer H., Spicher S., Bannwarth C., Grimme S. (2019). A generally applicable
atomic-charge dependent London dispersion correction. J. Chem. Phys..

[16] Goringe C. M., Bowler D. R., Hernández E. (1997). Tight-binding
modelling of materials. Rep. Prog. Phys..

[17] Butler P. W. V., Day G. M. (2023). Reducing overprediction of molecular
crystal structures
via threshold clustering. Proc. Natl. Acad.
Sci. U. S. A..

[18] So/rensen M. R., Voter A. F. (2000). Temperature-accelerated dynamics for simulation of
infrequent events. J. Chem. Phys..

[19] Sahil M., Sarkar S., Mondal J. (2023). Long-time-step molecular dynamics
can retard simulation of protein-ligand recognition process. Biophys. J..

[20] Dasgupta S., Lambros E., Perdew J. P., Paesani F. (2021). Elevating density functional
theory to chemical accuracy for water simulations through a density-corrected
many-body formalism. Nat. Commun..

[21] Kühne T. D., Iannuzzi M., Del Ben M., Rybkin V. V., Seewald P., Stein F., Laino T., Khaliullin R. Z., Schütt O., Schiffmann F., Golze D., Wilhelm J., Chulkov S., Bani-Hashemian M. H., Weber V., Borštnik U., Taillefumier M., Jakobovits A. S., Lazzaro A., Pabst H., Müller T., Schade R., Guidon M., Andermatt S., Holmberg N., Schenter G. K., Hehn A., Bussy A., Belleflamme F., Tabacchi G., Glöß A., Lass M., Bethune I., Mundy C. J., Plessl C., Watkins M., VandeVondele J., Krack M., Hutter J. (2020). CP2K: An electronic
structure and molecular dynamics software package - Quickstep: Efficient
and accurate electronic structure calculations. J. Chem. Phys..

[22] Miceli G., de Gironcoli S., Pasquarello A. (2015). Isobaric first-principles molecular
dynamics of liquid water with nonlocal van der Waals interactions. J. Chem. Phys..

[23] Sabatini R., Gorni T., de Gironcoli S. (2013). Nonlocal van
der Waals density functional
made simple and efficient. Phys. Rev. B.

[24] Miceli G., Hutter J., Pasquarello A. (2016). Liquid Water
through Density-Functional
Molecular Dynamics: Plane-Wave vs Atomic-Orbital Basis Sets. J. Chem. Theory Comput..

[25] Ruiz
Pestana L., Mardirossian N., Head-Gordon M., Head-Gordon T. (2017). Ab initio molecular dynamics simulations of liquid
water using high quality meta-GGA functionals. Chem. Sci..

[26] Goedecker S., Teter M., Hutter J. (1996). Separable
dual-space Gaussian pseudopotentials. Phys.
Rev. B.

[27] Hutter J. (2012). Car–Parrinello
molecular dynamics. Wiley Interdiscip. Rev.
Comput. Mol. Sci..

[28] VandeVondele J., Mohamed F., Krack M., Hutter J., Sprik M., Parrinello M. (2004). The influence
of temperature and density functional
models in ab initio molecular dynamics simulation of liquid water. J. Chem. Phys..

[29] Skinner L. B., Huang C., Schlesinger D., Pettersson L. G. M., Nilsson A., Benmore C. J. (2013). Benchmark oxygen-oxygen
pair-distribution
function of ambient water from x-ray diffraction measurements with
a wide Q-range. J. Chem. Phys..

[30] Hjorth
Larsen A., Jørgen Mortensen J., Blomqvist J., Castelli I. E., Christensen R., Dułak M., Friis J., Groves M. N., Hammer B., Hargus C., Hermes E. D., Jennings P. C., Bjerre Jensen P., Kermode J., Kitchin J. R., Leonhard Kolsbjerg E., Kubal J., Kaasbjerg K., Lysgaard S., Bergmann
Maronsson J., Maxson T., Olsen T., Pastewka L., Peterson A., Rostgaard C., Schiøtz J., Schütt O., Strange M., Thygesen K. S., Vegge T., Vilhelmsen L., Walter M., Zeng Z., Jacobsen K. W. (2017). The atomic
simulation environment-a Python library for working with atoms. J. Phys.: Condens. Matter.

[31] Holz M., Heil S. R., Sacco A. (2000). Temperature-dependent
self-diffusion
coefficients of water and six selected molecular liquids for calibration
in accurate 1H NMR PFG measurements. Phys. Chem.
Chem. Phys..

[32] Leimkuhler, B. ; Reich, S. Simulating Hamiltonian Dynamics; Cambridge Monographs on Applied and Computational Mathematics; Cambridge University Press, 2005, pp 234–256.

