# Supplementary material for: Accelerating Variable Cell Shape Molecular Dynamics with a Position-Dependent Mass Matrix
Source: J Chem Theory Comput. 2025 Jun 13;21(12):5827–37. doi: 10.1021/acs.jctc.5c00237 (PMC12199461; doi:10.1021/acs.jctc.5c00237)
Supplement: Supplementary file 1 [file ct5c00237_si_001.pdf]

Supporting Information: Accelerating variable cell shape molecular dynamics with a position-dependent mass matrix

Martin Sommer-Jörgensen,* Marco Krummenacher, and Stefan Goedecker

Department of Physics, University of Basel, Klingelbergstrasse 82, CH-4056 Basel, Switzerland

E-mail: martin.sommer@unibas.ch

May 26, 2025

S1 Numerical proof of the symplecticness of the variable cell shape MTMD integrator.

The integrator presented in Sec. 2 of the main article is symplectic, because it is constructed from the Hamiltonian equations of motions using the generalized leapfrog integrator. A numerical indicator of the symplectic characteristics is the excellent long-term energy conservation, shown in Fig. 5 of the main article. In this section, we present a numerical proof of the symplecticness by showing the conservation of the symplectic 2-form along the propagated trajectory. The propagation of the symplectic 2-form can be calculated from the Jacobian of the integrator. We consider a single integration step, starting from the phase-space coordinates $\boldsymbol{x} = (\boldsymbol{q}, \boldsymbol{p})$ and ending at the phase-space coordinates $\boldsymbol{X} = (\boldsymbol{Q}, \boldsymbol{P})$. The Jacobian is computed with finite differences, which involves choosing a finite difference step size h. An analytic Hessian matrix is not available for the DFTB+ calculator. We split the Jacobian into four equally sized blocks by distinguishing between the position and momentum subspaces. For the computation to be numerically conclusive, we need to treat the four blocks differently because they have different physical units and also quite different magnitudes. In particular, the derivatives with respect to the position coordinates have roughly 1000 times higher magnitudes than the derivatives with respect to momentum coordinates. For the same reason, we use a finite difference step size of $1000h$ when calculating the directional derivative along a momentum coordinate axis. We do not treat the derivatives

with respect to the lattice vectors specially because their magnitudes are not very different, although they have different units. The Jacobian is:

$$\frac{\partial \boldsymbol{X}}{\partial \boldsymbol{x}} = \begin{pmatrix} \frac{\partial \boldsymbol{Q}}{\partial \boldsymbol{q}} & \frac{\partial \boldsymbol{Q}}{\partial \boldsymbol{p}} \\ \frac{\partial \boldsymbol{P}}{\partial \boldsymbol{q}} & \frac{\partial \boldsymbol{P}}{\partial \boldsymbol{p}} \end{pmatrix} \tag{1}$$

The integrator is symplectic if and only if $\frac{\partial \boldsymbol{X}}{\partial \boldsymbol{x}}^T J \frac{\partial \boldsymbol{X}}{\partial \boldsymbol{x}} = J$, with $J = \begin{pmatrix} 0 & I \\ -I & 0 \end{pmatrix}$ the constant structure matrix of the given phase space coordinate system. This condition is equivalent to the following set of conditions in terms of matrix blocks:

$$\begin{aligned} \frac{dQ}{dq}^T \frac{dP}{dq} - \frac{dP}{dq}^T \frac{dQ}{dq} &= 0 \\ \frac{dQ}{dq}^T \frac{dP}{dp} - \frac{dP}{dq}^T \frac{dQ}{dp} &= I \\ \frac{dQ}{dp}^T \frac{dP}{dq} - \frac{dP}{dp}^T \frac{dQ}{dq} &= -I \\ \frac{dQ}{dp}^T \frac{dP}{dp} - \frac{dP}{dp}^T \frac{dQ}{dp} &= 0 \end{aligned} \tag{2}$$

The third equation can be discarded because it is equivalent to the second one. Next, we calculate the relative errors of the norms of the matrix-valued violations of the symplecticness conditions. We also include the relative error of the determinant in the analysis:

$$\begin{aligned} \epsilon_{QQ} &= \frac{\left\| \frac{dQ}{dq}^T \frac{dP}{dq} - \frac{dP}{dq}^T \frac{dQ}{dq} \right\|}{\left\| \frac{dQ}{dq}^T \frac{dP}{dq} \right\| + \left\| \frac{dP}{dq}^T \frac{dQ}{dq} \right\|} \\ \epsilon_{QP} &= \frac{\left\| \frac{dQ}{dq}^T \frac{dP}{dp} - \frac{dP}{dq}^T \frac{dQ}{dp} - I \right\|}{\left\| \frac{dQ}{dq}^T \frac{dP}{dp} \right\| + \left\| \frac{dP}{dq}^T \frac{dQ}{dp} \right\|} \\ \epsilon_{PP} &= \frac{\left\| \frac{dQ}{dp}^T \frac{dP}{dp} - \frac{dP}{dp}^T \frac{dQ}{dp} \right\|}{\left\| \frac{dQ}{dp}^T \frac{dP}{dp} \right\| + \left\| \frac{dP}{dp}^T \frac{dQ}{dp} \right\|} \\ \epsilon_{\det} &= \left| \det \frac{\partial \boldsymbol{X}}{\partial \boldsymbol{x}} - 1 \right| \end{aligned} \tag{3}$$

For both a symplectic and a non-symplectic integrator we expect that the errors are of some order in the time step, in the cases we ecountered $\mathcal{O}(\Delta t)$, since in the limit of small time steps an integration step becomes the identity map. The distinction is, however, that for a fixed Δt, the error of a symplectic integrator goes to zero when the step size for computing the finite differences Jacobian goes to zero (until noise comes into play), while for the non-symplectic integrator briefly mentioned in Sec. 1 of the main article, a constant error remains. This distinct behavior is made evident in Fig. S1.

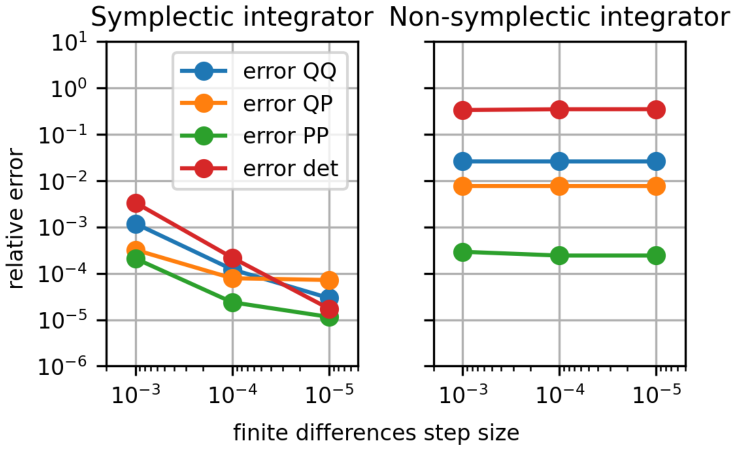

Figure S1: Relative errors of the symplecticness conditions.

We see that for the symplectic integrator, the relative error of the symplecticness conditions is proportional to the finite-difference step size. For a non-symplectic integrator, the relative error converges to a finite value. The difference is most pronounced for the relative error of the determinant, which describes the phase space volume conservation and which has a 4 orders of magnitude smaller error with the symplectic integrator. This finishes the numerical proof that the integrator is symplectic.

S2 Temperature-energy diagram of molecular nitrogen near the boiling point

The NPE-ensemble has pressure and energy (enthalpy) as external parameters and volume and temperature as observables. Here we give an example of a temperature-energy and a volume-energy diagram for molecular nitrogen near its boiling point. We have conducted 400 independent standard MD runs and 400 independent MTMD runs, each with 512 molecules and 100'000 time steps. The starting configuration had the density of liquid nitrogen. The time steps were 2 fs for standard MD and 10 fs for MTMD. The lattice vector mass W was set at 80 atomic units. The 400 runs corresponded to 400 equally spaced energy levels that were implemented by assigning different initial kinetic energies. Energy conservation was well maintained in all runs, with fluctuations in a range of 2 eV for standard MD and 0.5 eV for MTMD. Volumes and temperatures were calculated as an average of instantaneous volumes and temperatures of the second half of the runs. A transition time was measured whenever the volume had increased to about twice the equilibrated volume of the liquid, just enough such that the system did not return to the liquid phase. In Fig. S2, we can recognize the phase transition between liquid nitrogen and gas nitrogen. With increasing energy, the temperature increases first linearly. When the boiling temperature of nitrogen (experimentally 77 K) is reached, the temperature continues to rise, and the system enters a superheated state. At some point, there is a significant probability of evaporation, which is an event that involves a nucleation process. The temperature then drops and exhibits

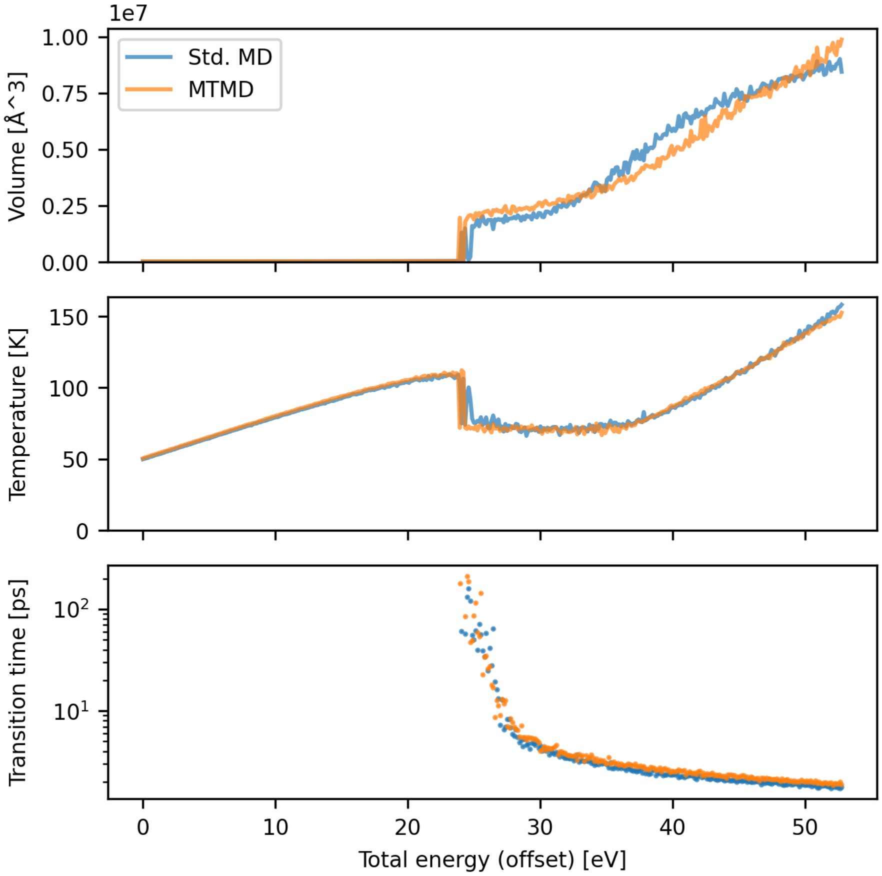

Figure S2: The volume-energy and temperature-energy diagram of nitrogen near its boiling point and the transition time. The simulations start in the liquid phase and there is an energy range in which superheating occurs without evaporation.

a plateau, where nitrogen in the liquid and in the gas phase coexist. When all nitrogen has evaporated, the temperature rises again. We did not observe any significant difference between the two methods in terms of the probability of transitioning from the superheated to mixed phase state within the limited number of simulation steps. However, we compared the transition times of both methods. We could see that the transition times of standard MD and MTMD at the same energy levels are similar, with the average transition time taken over the subset where both methods showed a transition being only 10% longer in MTMD. This suggests that the five-times longer time step of MTMD offers an advantage. In the gas phase, the volume shows a significant low-frequency oscillation, which is caused by the fact that the lattice vector mass W was optimized for the liquid phase and is too high for the gas phase, since the gas has a much higher compressibility. These oscillations could not be entirely neutralized by calculating the temporal averages of the final phase of the equilibration. The oscillations are more noticeable in standard MD than in MTMD because of the shorter simulation time.

S3 Derivative of the mass matrix in variable cell shape MTMD

In Sec. 2 of the main article, we have introduced the mass-weighted metric tensor G:

$$G(\boldsymbol{s}, \boldsymbol{h})_{ij} = \boldsymbol{h}^T M(\boldsymbol{s}, \boldsymbol{h})_{ij} \boldsymbol{h} \tag{4}$$

The Hamiltonian equations of motion of the variable cell shape MTMD contain the derivative of G with respect to the scaled positions of the atoms and with respect to the lattice vectors. But in Sec. 4 of the main article, the mass is constructed using Cartesian coordinates and not scaled coordinates. We assign a new name $\tilde{M}(\boldsymbol{R}, \boldsymbol{h})$ to the function that provides the mass matrix for given Cartesian coordinates $\boldsymbol{R}$, and express the mass matrix in scaled coordinates in terms of the mass matrix in Cartesian coordinates $M(\boldsymbol{s}, \boldsymbol{h}) = \tilde{M}(\boldsymbol{R}(\boldsymbol{s}, \boldsymbol{h}), \boldsymbol{h}) = \tilde{M}(\{\boldsymbol{h}\boldsymbol{s}_i\}, \boldsymbol{h})$. The transformation of the derivatives in Cartesian coordinates to the derivatives in scaled coordinates can be calculated with the chain rule:

$$\frac{\partial M(\boldsymbol{s}, \boldsymbol{h})}{\partial \boldsymbol{s}_i} = \sum_j \frac{\partial \tilde{M}}{\partial \boldsymbol{R}_j} \cdot \frac{\partial \boldsymbol{R}_j}{\partial \boldsymbol{s}_i} = \frac{\partial}{\partial \boldsymbol{s}_i} \left(\frac{\partial \tilde{M}}{\partial \boldsymbol{R}_i^T} \boldsymbol{h}\boldsymbol{s}_i \right) = \boldsymbol{h}^T \frac{\partial \tilde{M}}{\partial \boldsymbol{R}_i} \tag{5}$$

$$\frac{\partial M(\boldsymbol{s}, \boldsymbol{h})}{\partial \boldsymbol{h}} = \sum_i \frac{\partial \tilde{M}}{\partial \boldsymbol{R}_i} \cdot \frac{\partial \boldsymbol{R}_i}{\partial \boldsymbol{h}} + \frac{\partial \tilde{M}}{\partial \boldsymbol{h}} = \sum_i \frac{\partial \tilde{M}}{\partial \boldsymbol{R}_i} \boldsymbol{s}_i^T + \frac{\partial \tilde{M}}{\partial \boldsymbol{h}} \tag{6}$$

In this work, we further simplify the construction of the derivative with respect to $\boldsymbol{h}$ by putting all atoms of a single molecule into the same cell. This situation is also depicted in Fig. 2 of the main article, where no bonds are interrupted and continued on the other side of the unit cell. The interaction between bonded atoms then only depends on the position coordinates $\boldsymbol{R}$ and not on the lattice vectors $\boldsymbol{h}$ and the mass matrix function simplifies to $M(\boldsymbol{s}, \boldsymbol{h}) = \tilde{M}(\{\boldsymbol{h}\boldsymbol{s}_i\})$. By applying the product rule to Eq. 4, we can calculate the derivative

of the quadratic form $\boldsymbol{p}_{\boldsymbol{s}}^T G \boldsymbol{p}_{\boldsymbol{s}}$ with respect to $\boldsymbol{s}$:

$$\frac{\partial G_{ij}}{\partial \boldsymbol{s}_k} = \frac{\partial \left(\boldsymbol{h}^T M_{ij} \boldsymbol{h}\right)}{\partial \boldsymbol{s}_k} = \boldsymbol{h}^T \left(\boldsymbol{h}^T \frac{\partial \tilde{M}}{\partial \boldsymbol{R}_k}\right)_{ij} \boldsymbol{h} \tag{7}$$

$$\frac{\partial}{\partial \boldsymbol{s}_k} \sum_{ij} \boldsymbol{p}_{\boldsymbol{s},i}^T G_{ij} \boldsymbol{p}_{\boldsymbol{s},j} = \sum_{ij} \boldsymbol{p}_{\boldsymbol{s},i}^T \boldsymbol{h}^T \frac{\partial M_{ij}}{\partial \boldsymbol{s}_k} \boldsymbol{h} \boldsymbol{p}_{\boldsymbol{s},j} = \sum_{ij} \boldsymbol{p}_{\boldsymbol{s},i}^T \boldsymbol{h}^T \left(\boldsymbol{h}^T \frac{\partial \tilde{M}}{\partial \boldsymbol{R}_k}\right)_{ij} \boldsymbol{h} \boldsymbol{p}_{\boldsymbol{s},j} \tag{8}$$

Further, we calculate the derivative of the quadratic form $\boldsymbol{p}_{\boldsymbol{s}}^T G \boldsymbol{p}_{\boldsymbol{s}}$ with respect to $\boldsymbol{h}$:

$$\begin{aligned}
\frac{\partial}{\partial \boldsymbol{h}} \sum_{ij} \boldsymbol{p}_{\boldsymbol{s},i}^T G_{ij} \boldsymbol{p}_{\boldsymbol{s},j} =& \frac{\partial}{\partial \boldsymbol{h}} \sum_{ij} \boldsymbol{p}_{\boldsymbol{s},i}^T \boldsymbol{h}^T M_{ij} \boldsymbol{h} \boldsymbol{p}_{\boldsymbol{s},j} \\
=& \sum_{ij} \left(M_{ij} \boldsymbol{h} \boldsymbol{p}_{\boldsymbol{s},j} \boldsymbol{p}_{\boldsymbol{s},i}^T + \boldsymbol{p}_{\boldsymbol{s},i}^T \boldsymbol{h}^T \frac{\partial M_{ij}}{\partial \boldsymbol{h}} \boldsymbol{h} \boldsymbol{p}_{\boldsymbol{s},j} + M_{ij}^T \boldsymbol{h} \boldsymbol{p}_{\boldsymbol{s},i} \boldsymbol{p}_{\boldsymbol{s},j}^T \right) \\
=& 2 \sum_{ij} M_{ij} \boldsymbol{h} \boldsymbol{p}_{\boldsymbol{s},j} \boldsymbol{p}_{\boldsymbol{s},i}^T + \sum_{ij} \boldsymbol{p}_{\boldsymbol{s},i}^T \boldsymbol{h}^T \frac{\partial M_{ij}}{\partial \boldsymbol{h}} \boldsymbol{h} \boldsymbol{p}_{\boldsymbol{s},j} \\
=& 2 \sum_{ij} M_{ij} \boldsymbol{h} \boldsymbol{p}_{\boldsymbol{s},j} \boldsymbol{p}_{\boldsymbol{s},i}^T + \sum_{ij} \boldsymbol{p}_{\boldsymbol{s},i}^T \boldsymbol{h}^T \left(\sum_k \frac{\partial \tilde{M}_{ij}}{\partial \boldsymbol{R}_k} \boldsymbol{s}_k^T + \frac{\partial \tilde{M}_{ij}}{\partial \boldsymbol{h}} \right) \boldsymbol{h} \boldsymbol{p}_{\boldsymbol{s},j}
\end{aligned} \tag{9}$$

The derivative of the inverse of G is computed using the identity $\frac{\partial G^{-1}}{\partial x} = -G^{-1} \frac{\partial G}{\partial x} G^{-1}$.

We still need to provide the derivative of $\tilde{M}$ with respect to $\boldsymbol{R}$. The mass matrix is constructed from the Hessian matrix in internal coordinates in Eq. 22 of the main article and is composed of an unscaled matrix $\tilde{M}_u$ and a factor c which scales the mass matrix such that the determinant of the mass matrix is constant:

$$\tilde{M}(\boldsymbol{R}) = \tilde{M}_u(\boldsymbol{R}) c(\boldsymbol{q}). \tag{10}$$

This defines $c(\boldsymbol{R})$ up to a multiplicative constant as $c \propto \det\left(\tilde{M}_u\right)^{-1/3N}$. The derivative of $\tilde{M}$ can be calculated with the product rule:

$$\begin{aligned}
\frac{\partial \tilde{M}(\boldsymbol{R})}{\partial \boldsymbol{R}_k} \propto& \frac{\partial \tilde{M}_u(\boldsymbol{R})}{\partial \boldsymbol{R}_k} \det\left(\tilde{M}_u\right)^{-1/3N} + \tilde{M}_u \frac{\partial \det\left(\tilde{M}_u\right)^{-1/3N}}{\partial \boldsymbol{R}_k} \\
=& \frac{\partial \tilde{M}_u(\boldsymbol{R})}{\partial \boldsymbol{R}_k} \det\left(\tilde{M}_u\right)^{-1/3N} - \tilde{M}_u \frac{\det\left(\tilde{M}_u\right)^{-1/3N}}{3N} \mathrm{Tr}\left(\tilde{M}_u^{-1} \frac{\partial \tilde{M}_u}{\partial \boldsymbol{R}_k} \right)
\end{aligned} \tag{11}$$
